# 2133. Surveillance of Eravacycline Against Gram-positive Clinical Pathogens, Including Resistant Isolates, Collected Worldwide From Multiple Infection Sites During 2021

**DOI:** 10.1093/ofid/ofad500.1756

**Published:** 2023-11-27

**Authors:** Stephen Hawser, Nimmi Kothari, Federica Monti, Tony Hodges, Kristie Zappas

**Affiliations:** IHMA Europe, Monthey, Valais, Switzerland; IHMA, Monthey, Valais, Switzerland; IHMA, Monthey, Valais, Switzerland; La Jolla Therapeutics, Waltham, Massachusetts; Innoviva Specialty Therapeutics, Inc., Waltham, Massachusetts

## Abstract

**Background:**

Eravacycline is a fully synthetic, fluorocycline approved for the treatment of complicated intra-abdominal infections (cIAI) in patients ≥18 years of age in Europe, Singapore, Hong Kong, mainland China, the US, and the UK. Previous surveillance studies of eravacycline have demonstrated potent *in vitro* activity against specific Gram-positive pathogens. The purpose of this study was to further monitor the *in vitro* activity of eravacycline against *Staphylococcus aureus* (including methicillin-resistant *S. aureus*, MRSA), *Enterococcus* spp. (including vancomycin-resistant *Enterococcus*, VRE) and *Streptococcus* spp.

**Methods:**

A total of 1,557 clinical isolates were collected worldwide during 2021 from multiple infection sources, including bodily fluids, gastrointestinal, genitourinary, and respiratory. Of the 1,557 isolates, 316, 649, and 592 were from Asia/Pacific, Europe, and North America, respectively. Minimum inhibitory concentrations (MICs) were determined by CLSI broth microdilution. For comparative purposes, antibiotic susceptibility for eravacycline and tigecycline was determined with both FDA and EUCAST breakpoints, where available.

**Results:**

Summary MIC data for eravacycline and tigecycline, as a selected comparator, are shown in the Table. Susceptibility to eravacycline in all Gram-positive pathogens was greater than 90% for most species/phenotypes though was notably higher when EUCAST breakpoints were applied to the analysis.

**Figure:**
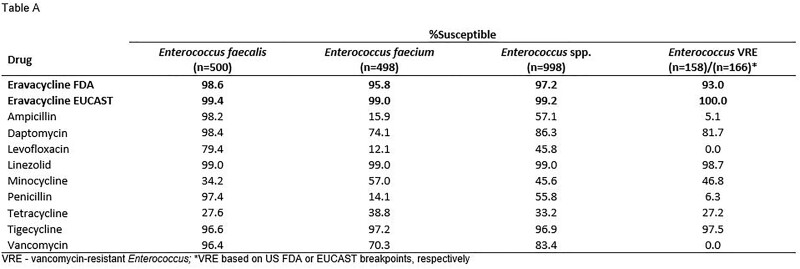

**Figure:**
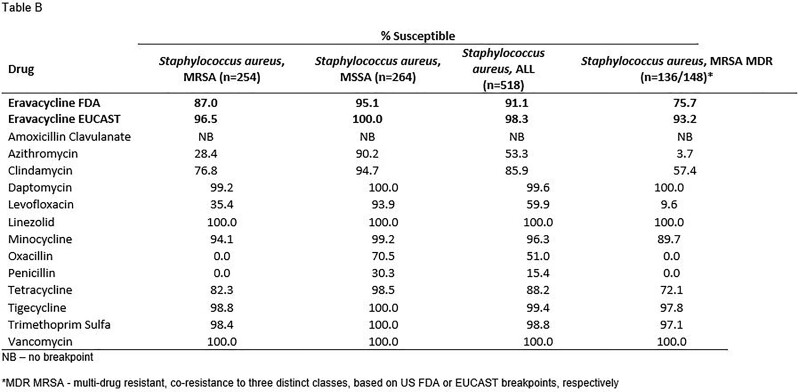

**Figure:**
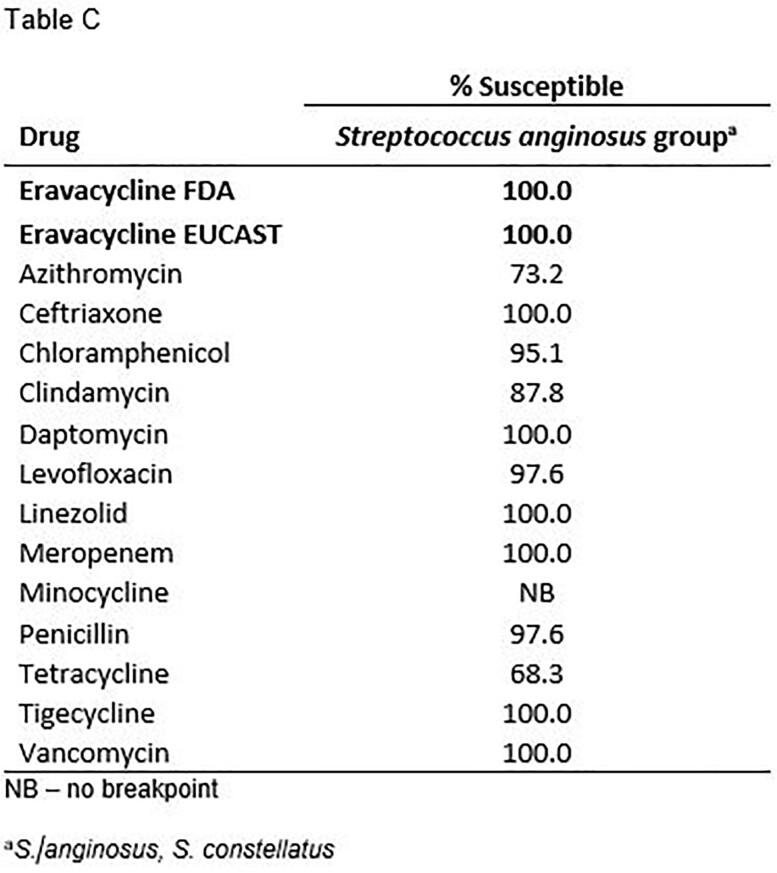

**Conclusion:**

Eravacycline demonstrated sustained high susceptibility rates against clinically important Gram-positive pathogens, including resistant isolates. Importantly, no CLSI breakpoint exists to date for eravacycline, though the use of EUCAST breakpoints showed higher susceptibilities compared with the use of FDA breakpoints. Harmonization of clinical breakpoints for eravacycline would be desirable.

**Disclosures:**

**Stephen Hawser, PhD**, Allecra: study funding|Innoviva Specialty Therapeutics, Inc.: Honoraria|Roche: Honoraria|Roche: This project has been funded by BARDA (HHSO100201600038C). **Nimmi Kothari, PhD**, Allecra: Allecra (study funding)|Innoviva Specialty Therapeutics, Inc.: Honoraria|Roche: Honoraria|Roche: This project has been funded by BARDA (HHSO100201600038C). **Federica Monti, PhD**, Allecra: Study funded|Innoviva Specialty Therapeutics, Inc.: Honoraria **Tony Hodges, MD**, La Jolla Pharmaceutical Company: Employee|La Jolla Pharmaceutical Company: Stocks/Bonds **Kristie Zappas, PhD**, La Jolla Pharmaceutical Company: employee|La Jolla Pharmaceutical Company: Stocks/Bonds

